# S1 Radiculopathy Initially Presenting With Sole Knee Flexion Weakness: A Case Report

**DOI:** 10.7759/cureus.57673

**Published:** 2024-04-05

**Authors:** Stavros Stamiris, Christos Karampalis, Dimitrios Stamiris, Elissavet Anestiadou, Pavlos Christodoulou

**Affiliations:** 1 Department of Orthopaedics, 424 General Military Hospital, Thessaloniki, GRC; 2 4th Surgical Department, Papanikolaou General Hospital of Thessaloniki, Aristotle University of Thessaloniki, Thessaloniki, GRC

**Keywords:** atypical neurological presentation, lumbar disc hernia, hamstrings weakness, rare case report, s1 radiculopathy

## Abstract

Lumbar disc herniation can lead to low back pain and/or sciatica, as well as manifest with neurological symptoms in specific dermatomal/myotomal patterns due to nerve root irritation. S1 radiculopathy is the result of L5-S1 disc herniation and is usually presented with foot plantar flexion/eversion weakness and hypoesthesia in the lateral aspect of the foot. We present a case of S1 radiculopathy that initially presented with hamstring weakness and posterior knee pain as the only manifestations, leading to a delay in the initial diagnosis and treatment. To the best of our knowledge, no previous studies have reported this atypical presentation that resulted from S1 radiculopathy. This case report is of great clinical value, as it will help diagnosticians broaden the diagnostic range in patients with similar symptomatology and avoid diagnostical pitfalls.

## Introduction

Lumbar disc herniation (LDH) is a degenerative disease of the spine that affects 1-3% of the population annually and can present with low back pain and/or sciatica due to nerve root irritation [1,2]. These symptoms can be accompanied by neurological manifestations like motor and sensory deficits in specific myotomal and dermatomal patterns [3].

Most cases of LDH involve the L4-L5 and L5-S1 discs and can be manifested with neurological symptoms that are the result of compression of the L4 root (L4-L5 foraminal disc herniation), L5 root (L4-L5 paracentral or L5-S1 foraminal disc herniation), or S1 root (L5-S1 paracentral disc herniation) [4].

More specifically, S1 radiculopathy is the result of L5-S1 paracentral disc herniation. Neurological examination may reveal foot plantar flexion and eversion weakness (gastrocnemius-soleus and peroneal muscles, respectively), decreased or diminished Achilles tendon reflex, dermatomal pain in the posterior leg, as well as sensory deficit on the lateral posterior calf and lateral foot area.

We report a rare case of S1 LDH that presented with knee flexion weakness and normal foot plantar flexion/eversion muscle strength. To the best of our knowledge, this rare manifestation of S1 radiculopathy has not been reported.

This case report is presented in accordance with Surgical CAse REport (SCARE) guidelines [5].

## Case presentation

A 43-year-old woman presented to the outpatient orthopedic office of our tertiary military hospital, after a referral from a family doctor, complaining of pain in the posterior aspect of her left leg, from mid-thigh to mid-calf. The onset of symptoms was gradual, without a specific traumatic event, and they persisted for 10 days prior to consultation. The pain was exacerbated with physical activities or prolonged sitting. No relevant medical history was reported. The patient’s occupation involved manual laboring, including handwork, frequent lifting of objects, and kneeling.

Examination of her left hip (including the FAIR (flexion, adduction, and internal rotation) test for piriformis syndrome) and left knee was unremarkable. Laseque’s sign and Braggard's sign were absent on clinical examination. There was no evidence of hypoesthesia in her left lower extremity. Motor examination revealed weakness in knee flexion 4/5 on the Medical Research Council (MRC) muscle strength scale [6]. Spine and hip X-rays were unremarkable. Knee X-rays revealed mild osteoarthritic changes not indicative of our clinical findings. The patient was treated with non-steroidal anti-inflammatory drugs (NSAIDs) (etorecoxib 60 mg twice a day) and a left knee and lumbar spine MRI were ordered.

**Figure 1:**
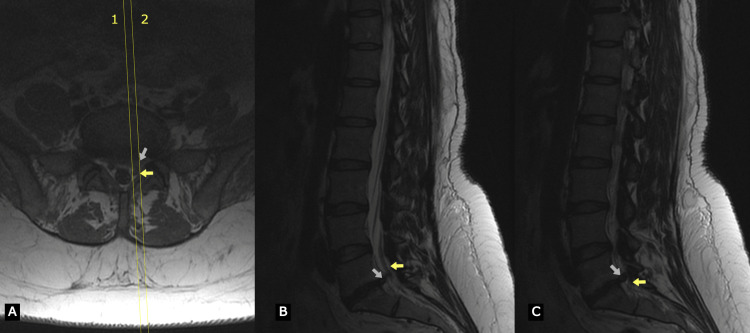
T2 weighted images of lumbar spine MRI that present a left paracentral disc herniation resulting in compression of the left S1 root Marks 1 and 2 in image A correspond to images B and C, respectively. The white arrow indicates disc herniation while the yellow arrow indicates S1 root.

On reexamination five days later, the clinical evaluation revealed a positive tension sign (positive Laseque sign at 40 degrees) on her left leg, reduced muscle power of knee flexion and foot plantar flexion (4/5 on MRC), as well as reduced sensation on the lateral side of her foot (S1 dermatomal distribution). Her knee MRI was unremarkable, but lumbar spine MRI revealed a large paracentral disc bulge at the L5-S1 level resulting in compression of the descending S1 nerve root on the left side as seen in Figure 1. The patient was treated with a 14-day per os corticosteroid regimen (prednisolone, initially 20 mg twice a day for five days followed by gradual tapering over a period of nine days) and physical therapy.

The patient was reevaluated after the conclusion of cortisone treatment; no improvement was noted regarding her pain severity or neurological status. Upon these findings, the patient was admitted to the orthopedic department of our hospital and a L5-S1 microdiscectomy was scheduled. Intraoperatively, the prolapsed part of the L5-S1 intervertebral disc was gently removed from the lateral recess and some free parts of the degenerated disc were removed through the annulus fibrosus. There were no complications during surgery. Pain was completely resolved within 24 hours postoperative and the patient was discharged home and returned for follow-up visits at regular intervals to the outpatient orthopedic office. Complete resolution of symptoms was obtained within four weeks after surgery.

## Discussion

The L5-S1 segment is the second most common site of lumbar disc herniation following L4-L5. The most typical presentation of S1 radiculopathy in terms of neurological manifestation involves buttock and posterior leg pain, sensory deficit in the lateral-posterior leg and lateral foot, gastrocnemius-soleus complex weakness, and decreased or absent Achilles tendon reflex [3,7,8].

Despite the aforementioned typical presentation, it is established in the literature that the S1 nerve root contributes to the innervation of hamstring muscles. Specifically, the semimembranosus, semitendinosus, and long head of the biceps femoris are innervated by the tibial division of the sciatic nerve (L5, S1, S2), while the short head of the biceps femoris is innervated by the peroneal division of sciatic nerve (L5, S1, S2).

Sharrard et al. performed intraoperative root stimulation in 41 infants treated for myelomeningocele to examine the effect on lower limb muscles. They reported that stimulation of the S1 root caused, among others, strong knee flexion attributed to the biceps femoris, semimembranosus, and semitendinosus activation [9]. Liguori et al. used electrodiagnostic studies in 27 subjects by stimulating the spinal nerves and recording the evoked responses from specific lower limb muscles, to evaluate motor innervation of muscles of lower limb. They reported that biceps femoris evoked a response after stimulation of the L5 and S1 roots [10]. Tsao et al. compared surgical and preoperative electrodiagnostic findings in 45 patients with surgically verified, single-level spinal nerve root lesions to determine muscles that are affected by specific single-level lumbosacral radiculopathy. They concluded that biceps femoris (both short and long heads) motor compromise is a typical finding in S1 radiculopathy but not semitendinosus [11].

In our case, the patient presented with an atypical initial clinical manifestation that involved no low back pain, negative tension signs, pain limited in the posterior surface of the knee, normal sensory output, and isolated weakness in knee flexors with normal gastrocnemius-soleus and peroneal muscle strength. The latter is of importance because knee flexors are usually not included in the examination when testing lumbosacral myotomes. This case report is of value because it reports an unusual initial presentation of S1 radiculopathy that can lead to a delay in diagnosis or diagnostical pitfalls. It aims to raise clinical suspicion and broaden the diagnostic range in patients with S1 radiculopathy and atypical clinical presentation.

## Conclusions

Hamstring weakness is a possible manifestation of S1 radiculopathy and can, in some rare cases, be the only neurological presentation. A high index of clinical suspicion is required from physicians in order to avoid early diagnostical pitfalls or delays in diagnosis.
